# Effect of local and systemic administration of atorvastatin for improving bone healing on critical defects

**DOI:** 10.1590/0103-6440202406114

**Published:** 2024-10-25

**Authors:** Fábio Vieira de Miranda-Filho, Stéfany Barbosa, Olavo Alcalde Panigali, Mirela Caroline Silva, Monique Gonçalves da Costa, Franciele da Silva Flores, Edilson Ervolino, Letícia Helena Theodoro, Osvaldo Magro-Filho, Leonardo Perez Faverani

**Affiliations:** 1 Department of Diagnosis and Surgery. Sao Paulo State University-Unesp. Aracatuba School of Dentistry, Sao Paulo16015-050, Brazil; 2 Department of Basic Sciences. Sao Paulo State University-Unesp. Aracatuba School of Dentistry, Sao Paulo 16015-050, Brazil

**Keywords:** Osteogenesis, atorvastatin, bone regeneration

## Abstract

This study aimed to evaluate the impact of atorvastatin, administered both locally and systemically, on critical defects in the calvaria of rats. Thirty-six adult rats were randomly assigned to three groups, with all bone defects covered by a collagen membrane. The groups received different treatments: distilled water (GAD), where membranes were soaked in distilled water; systemic application of atorvastatin (GAS) at a dosage of 3.6mg/kg/day through gavage; and local application of atorvastatin (GAL). After 14 and 28 days, all animals were euthanized, and various assessments were conducted, including histometric analysis, measurement of linear residual defect, evaluation of newly formed bone area, determination of membrane and soft tissue area, cell count, and immunohistochemical analysis. Group GAS exhibited a significant reduction in residual defect compared to the other groups (p<0.05) and a lower number of osteocytes (p<0.05) in comparison with other groups. On day 28, both GAL and GAS groups showed a higher number of inflammatory cells compared to GAD (p<0.05). Immunolabeling of CD31 was similar for both groups, but in the case of osteocalcin, there was a significant increase in labeling for groups GAS and GAL between days 14 and 28 postoperative (p<0.05). In conclusion, systemic atorvastatin demonstrated enhanced osteogenesis in critical calvaria defects in rats, suggesting its efficacy in promoting bone regeneration without exerting a notable anti-inflammatory effect.

## Resumo

Este estudo teve como objetivo avaliar o impacto da atorvastatina, administrada tanto local quanto sistemicamente, em defeitos críticos na calvária de ratos. Trinta e seis ratos adultos foram randomicamente divididos em três grupos, com todos os defeitos ósseos cobertos por uma membrana de colágeno. Os grupos receberam diferentes tratamentos: água destilada (GAD), onde as membranas foram embebidas em água destilada; aplicação sistêmica de atorvastatina (GAS) numa dose de 3,6mg/kg/dia por gavagem; e aplicação local de atorvastatina (GAL). Após 14 e 28 dias, todos os animais foram sacrificados, e várias análises foram conduzidas, incluindo análise histométrica, medição do defeito residual linear, avaliação da área de osso recém-formado, determinação da área de membrana e tecido mole, contagem de células e análise imunohistoquímica. O grupo GAS apresentou uma redução significativa no defeito residual em comparação com os outros grupos (p<0.05) e um menor número de osteócitos (p<0.05) em comparação com os outros grupos. No dia 28, os grupos GAL e GAS exibiram um maior número de células inflamatórias em comparação com o GAD (p<0.05). A imunomarcação de CD31 foi semelhante para ambos os grupos, mas no caso da osteocalcina, houve um aumento significativo na marcação para os grupos GAS e GAL entre os dias 14 e 28 pós-operatórios (p<0.05). Em conclusão, a atorvastatina sistêmica demonstrou uma osteogênese aprimorada em defeitos críticos na calvária de ratos, sugerindo sua eficácia na promoção da regeneração óssea sem exercer um efeito anti-inflamatório notável.

**Figure:**
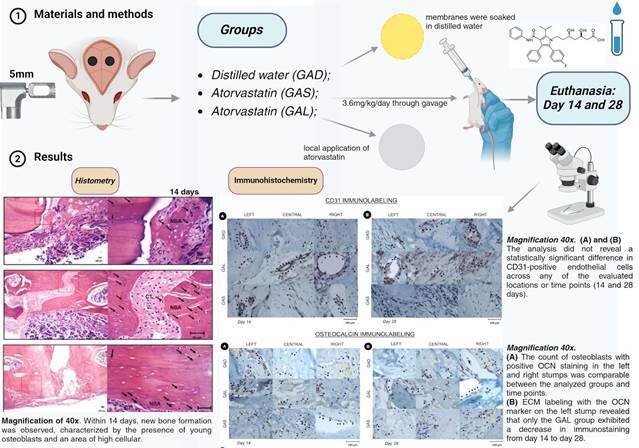

## Introduction

The repair of critical bone defects is a well-covered topic in the medical and dental literature.^1^ To reduce morbidity and discomfort in patients undergoing bone reconstruction, current studies are looking for materials that have osteogenic, osteoinductive and osteoconductive properties, which favor the repair of bone tissue or can act as an ideal bone substitute.^1^ ^,^ ^2^ The bone remodeling process consists of four sequential phases: 1) the activation phase, in which osteoclast progenitors are recruited to the damaged bone surface; 2) the resorption phase, when mature osteoclasts resorb damaged bone; 3) the reversion phase, in which osteoclasts die and osteoblast progenitors are recruited; and 4) the formation phase, when mature osteoblasts produce new bone matrix called osteoid, which is then mineralized.(3)

After tooth extraction, bone lesion removal, or maxillofacial traumas, significant alveolar bone remodeling can lead to a critical-size defects, necessitating bone reconstruction before oral rehabilitation with dental implants^4^ ^,^ ^5^. Although several materials have been used for these reconstructions, strategies to enhance osteogenic properties are necessary, especially for patients with bone metabolism alterations such as diabetes, hypertension, osteoporosis, malnutrition, and hypercholesterolemia^6^ ^,^ ^7^ ^,^ ^8^.

Studies have shown that Bone Morphogenetic Proteins (BMPs) are crucial in bone remodeling, being present in both endochondral and intramembranous formation, in addition to stimulating cell differentiation, the formation of cartilaginous matrix, and contributing to the chemotaxis process.^9^ ^,^ ^10^ Changes in BMP concentrations have been observed in studies showing that statins can increase BMP protein levels, thereby promoting bone formation.^10^ ^,^ ^11^

Statins (pravastatin, simvastatin, fluvastatin, atorvastatin, cerivastatin, pitavastatin and rosuvastatin) are lipoproteins that inhibit 3-hydroxy-3-methylglutaryl coenzyme A (HMG-CoA) reductase. They block cholesterol biosynthesis in liver cells, resulting in a greater captation and clearance of atherogenic LDL cholesterol (LDL-C) from the blood. Consequently, statins reduce the risk of developing atherosclerosis and its acute complications, such as acute myocardial infarction and ischemic stroke. In addition to reducing blood cholesterol levels, they act as anti-inflammatories, antioxidants, antibacterials and affect osteogenesis and angiogenesis.^12^ Mundy et al.^13^, and Ayukawa et al.^1^, have shown that other beneficial effects of statins include signaling and/or stimulation of BMP-2 and osteoblastic differentiation, which indicates an osteogenic potential. These findings have drawn the attention to new research in bone regeneration area.^1^ ^,^ ^13^

Tanabe et al.^14^, evaluated the local osteogenic effects of fluvastatin incorporated into biodegradable gelatin in rats, using micro-Ct and histology parameters. They concluded that biodegradable gelatin containing fluvastatin promoted osteogenesis in rat calvaria, revealing the potential of this method in regeneration studies.^15^ Furthermore, Yue et al.^16^, evaluated, in an in vivo study using critical defects in rabbit skulls, the use of locally administered simvastatin with BioOss®. Using histology, immunohistochemistry and fluorescence, they suggested that the local use of simvastatin significantly improves bone regeneration in the initial stages of healing.^15^ Mundy et al.^13^, carried out both in vitro and in vivo studies, using calvarial bone culture from rat pups (lovastatin, simvastatin, fluvastatin and mevastatin) and subcutaneous injections in rats (lovastatin and simvastatin), respectively. They observed an increase in bone neoformation when applying simvastatin and lovastatin and pointed out that these statins can promote the differentiation of osteoblasts by stimulating BMP-2.^3^ ^,^ ^13^

Lipophilic statins, particularly simvastatin and atorvastatin, are the most investigated statins and have shown good results in bone consolidation and renewal. Some studies have used atorvastatin in animals to assess bone regeneration and metabolism.^15^ ^,^ ^17^ ^,^ ^18^ Hong et al., demonstrated that systemic administration of atorvastatin increases trabecular bone mass, promotes bone formation in mice, and that its mechanism can assist in osteogenesis^17^ Nevertheless, local statin administration and statin therapy for repair is still being researched in vivo.^18^ Oryan et al., have shown that there are controversies regarding the dose, administration route and the effects of this therapy. It is therefore necessary to develop long-term studies and future clinical trials to establish a relationship between administration options and bone repair and regeneration.^18^ Additionally, most statins have minimal side effects, which supports their systemic and local use.^19^

The decision to use atorvastatin in this proposal is based on its increasing use in the clinical treatment of hypercholesterolemia and its lower side effects compared to other statins.^19^ For local application, despite statins having a low molecular weight, atorvastatin has better solubility, enabling its release into the tissue for bone regeneration and requiring further biological investigations.^20^

Therefore, this study has evaluated the effect of atorvastatin, applied locally and systemically, on critical defects in rat calvaria.

## Materials and methods

### Animals/Sample size

Thirty-six adult adult (N=36 defects) Wistar rats with body weight between 400 and 450g were used to carry out this study. The animals were kept in cages in an air-conditioned environment (4 per cage). They were fed standard solid food and water “ad libitum” throughout the experiment. The research project received approval by the Ethics Committee on the Use of Animals of the Araçatuba School of Dentistry - Unesp, Brazil (#00161-2017).

For the power test, the "Sample Size for ANOVA" tool in SigmaPlot 12.0 (Systat Software Inc., San Jose) was used, based on previous studies. Considering newly formed bone as the primary outcome, using the mean difference and standard deviation between two different groups, a minimum sample size of 6 defects per group and period was necessary to achieve a statistical power of 90%.^21^ ^,^ ^22^

### Anesthesia and surgical procedure

After fasting for 12 hours pre-operatively without water restriction, the animals were sedated with an intramuscular administration of Ketamine (70 mg/kg) and Xylazine (5 mg/kg). Following anesthetic induction and antisepsis of the treatment region, a trichotomy was performed in the fronto-parietal region, with the animals in a prone position.

Surgical access was performed, and the parietal bone was exposed on both sides. A surgical trephine with an internal diameter of 4 mm and an external diameter of 5 mm was used to perform the osteotomy in the median region between the parietals and the internal cortex. The osteotomized parietal bone was removed and the dura mater was kept intact, leaving a critical size defect (CSD) measuring 5 mm in diameter. The tissues were then sutured.

The CSDs in all animals were covered with collagen membrane (Bio-Gide® membrane: Geistlich Resorbable Natural Porcine Collagen Membrane, Wolhusen, Switzerland) and filled with clot.

The animals were randomized into three experimental groups (n=12) according to the therapy used: GAD (Bio-Gide® membrane soaked with distilled water), GAL (Bio-Gide® membrane soaked with a solution of 4.3mg of atorvastatin dissolved in 10mL of distilled water)^14^ and GAS (Bio-Gide membrane associated with systemic atorvastatin administration by gavage at a dose of 3.6 mg/kg/day).^20^

Euthanasia was performed after 14 and 28 days postoperatively.

### Euthanasia and preparation of samples for microscopic analysis

In each postoperative period, 6 animals from each group were euthanized by anesthetic overdose. The calvaria were removed and fixed in 10% buffered formalin solution (Analytical Reagents; Dental Dinâmica Ltda, Catanduva, Brazil) for 48 hours and then rinsed in running water for 24 hours.

The area of the original surgical defect and surrounding tissues were removed in bloc. Following decalcification in 10% EDTA solution, each specimen was divided longitudinally and processed for paraffin inclusion. Semi-serial sections of 5 µm were obtained in the longitudinal direction, after which the histological slides with even numbers were stained with Hematoxylin and Eosin (HE) and the odd ones submitted to immunohistochemical analysis.

### Histometry

The sections were mounted on histological slides and fixed with a coverslip and Permont resin to be photomicrographed under an optical microscope (Nikon® eclipse E100) coupled to a camera (Biovera® IS500). The images were obtained at 4x, 10x, 40x and 100x magnification. The lowest magnification was performed to create panoramic images of the defect, followed by enlarged images (10x, 40x, and 100x) in the central portion of the critical defect and on the sides, in addition to areas of the right and left stumps to capture HE staining and immunohistochemistry.

### Residual linear defect

The captured images were also recorded in a TIF file, followed by the assembly of panoramic images in Photoshop CC 2019 version, and later analysis was performed by the IMAGE J (Image Analyzer Program, Ontario, Canada) program to determine the histometric calculation. For this, the program was calibrated using the “set scale” tool, using pixels as a standard measure. Then, the images were opened in the program, and using the “straight” tool, measurement was performed in a linear manner.

### Membrane and soft tissue area

Images were captured with ISListen 3.2. They were recorded in a TIF file and analyzed using IMAGE J software (Image Analyzer Program, Ontario, Canada). For the histometric calculation of the membrane and soft tissue area in the region of the critical defect, the program was initially calibrated to use pixels as a standard measurement, in the “set scale” tool. Subsequently, the images were opened in the program and, using the “freehands” tool, the area in pixels was calculated and any separate areas were joined.

### Area of neoformed bone

For the histometric calculation of the area of neoformed bone in the region of the critical defect, the same methodology used for calculating the area of the membrane and soft tissue was applied.

### Cell count

Cells were counted using the images at 1000x^23^ magnification of HE stained slides. Images were captured in three locations^24^; left stump, central area of the defect and right stump. Next, inflammatory cells, osteoblasts, fibroblasts and osteocytes were counted. To count these cells, the “crosses'' tool in IMAGE J was used at 10,000 pixels per area, totaling 494 “crosses'' per image.

### Immunohistochemistry

Immunohistochemical reactions were performed on deparaffinized tissue. After this process and tissue treatment with solutions suitable for epitope recovery, the histological sections were incubated with a panel of polyclonal antibodies CD31 Lot A0649 (Biorbyt®) and OSTEOCALCIN (OCN) Lot BS7110 (Biorbyt®). These proteins were chosen to evaluate cellular responses related to vascularization and bone mineralization, respectively. Amplification was carried out using polymers in a fully automated process on a Ventana BenchMark GX device. Revealing was done with an UltraView DAB kit Lot F07797-F07855- (HRP Multimer. serial number: 1452607, Chromogen, serial number: 1497597, Cooper. serial number: 1454240, Inhibitor. serial number: 1467415 and H2O2. number serial number 1465306).

The immunohistochemical reactions were quantitatively evaluated by counting marked cells^24^. The images were captured with the same protocol used for counting cells in histometry, at the following three locations^24^: left stump of the defect, central area and right stump.

### CD31

The endothelial cells stained positively for the CD31 antibody were quantified.

### Osteocalcin (osteoblasts)

Using OCN antibodies, osteoblastic and pre-osteoblastic cells were identified and counted.

### Osteocalcin (ECM)

A semi-quantitative analysis was performed to measure the Extracellular Matrix (ECM). For this analysis SCORE immunostaining^24^ was used, ranging from 0 to 4 in ascending order according to the level of ECM labeling and cellular immunoreaction. The analysis was performed by a previously trained and calibrated examiner. The data collected through histometry and immunohistochemistry were grouped into a spreadsheet and submitted to statistical analysis.

### Statistical analysis

The statistical analyses were performed using SigmaPlot 12.0 (Exakt graph and Data Analysis, San Jose, CA, USA) at a significance level of 5%.

All quantitative parameters analyzed in this research were tested for normality using the Shapiro-Wilk test, which indicated homogeneity (p> 0.05). Subsequently, the Two-way ANOVA test was applied, followed by the Holm-Sidak post-test when p<0.05.

## Results

### Histological analysis

**Figure 1:**
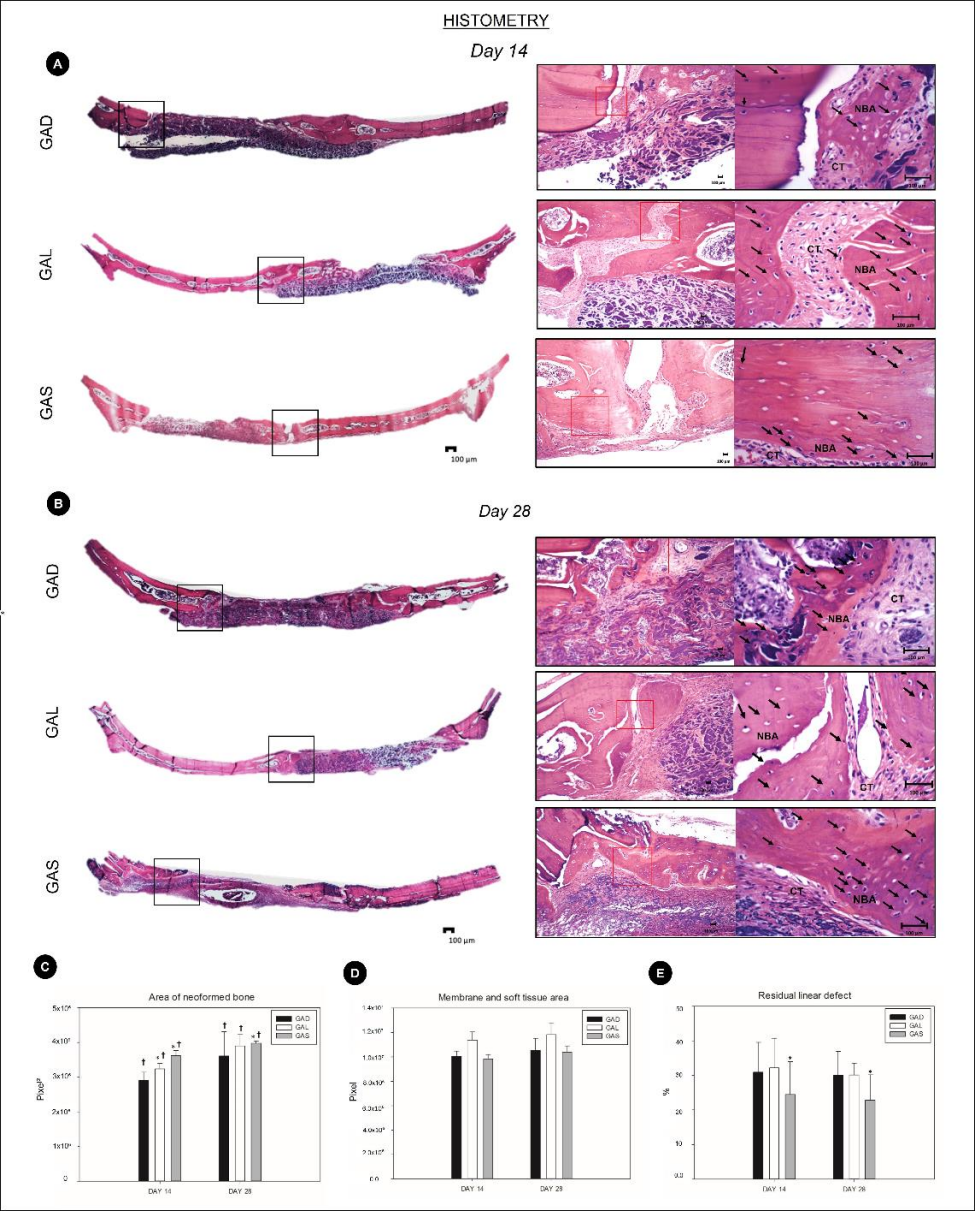
(A) Histological scheme showing panoramic images at 4x magnification, enlargement of the selected area at 10x (black zoom area), and further magnification at 40x (red zoom area), with H&E staining on day 14. (B) Histological scheme showing panoramic images at original 4x magnification, enlargement of the selected area at 10x (black zoom area), and further magnification at 40x (red zoom area), with H&E staining on day 28. Black arrows indicating new osteocytes (C) Graphical representation of area of neoformed bone in the experimental groups GAD, GAL, and GAS at 14 and 28 days postoperatively. *GAL and GAS showed increasing values at 14 days (p<0.05); GAS showed increasing values at 28 days (p<0.05). (D) Graphical representation of membrane and soft tissue area in the experimental groups GAD, GAL, and GAS at 14 and 28 days postoperatively. (E) Graphical representation of Residual Linear Defect in the experimental groups GAD, GAL, and GAS at 14 and 28 days postoperatively. *GAS at both 14 and 28 days showed increased values (p<0.05).

The histological analysis is presented in the general picture of the panoramic images (Figure 1-A, B) at 4x magnification to assess the defect and presence of stumps. In the histological analysis at magnifications of 4x, 10x and 40x over a period of 14 days, the area close to the edge of the defect (Figure 1-A), neoformed bone was noted, with young osteoblasts and an area of great cellularity, with emphasis on the group GAS.

On day 28, the presence of atorvastatin revealed a greater quantity of active osteoblasts and osteocytes, altering the quality of the bone tissue and presenting more mature areas (GAL). After systemic atorvastatin application (GAS) a small change in the tissue was noted, with a greater quantity of bone tissue and mineralized tissue (Figure 1- B).

### Area of neoformed bone

For the quantitative data, all groups showed an increase in the area of neoformed bone from 14 days to 28 days postoperative (p<0.05). In the intragroup comparison, on day 14, groups GAD and GAL were similar (p>0.05), and they demonstrated a lower area of neoformed bone compared to GAS (p<0.05). On the other hand, on day 28, group GAL exhibited an increased area of neoformed bone, being similar to GAS (p>0.05), and both showed higher values compared to GAD (p<0.05) (Figure 1-C).

### Membrane and soft tissue area

Membrane degradation was not influenced by atorvastatin, which demonstrated gradual degradation over time, with no difference in terms of the presence of atorvastatin or how it was applied (p>0.05)(Figure 1-D).

### Residual Linear Defect

For both the 14-day and 28-day experimental periods, groups GAD and GAL demonstrated comparable linear residual defect values (p>0.05), which were significantly higher than those observed in group GAS (p<0.05) (Figure 1 - E).

### Cellular quantification

**Figure 2:**
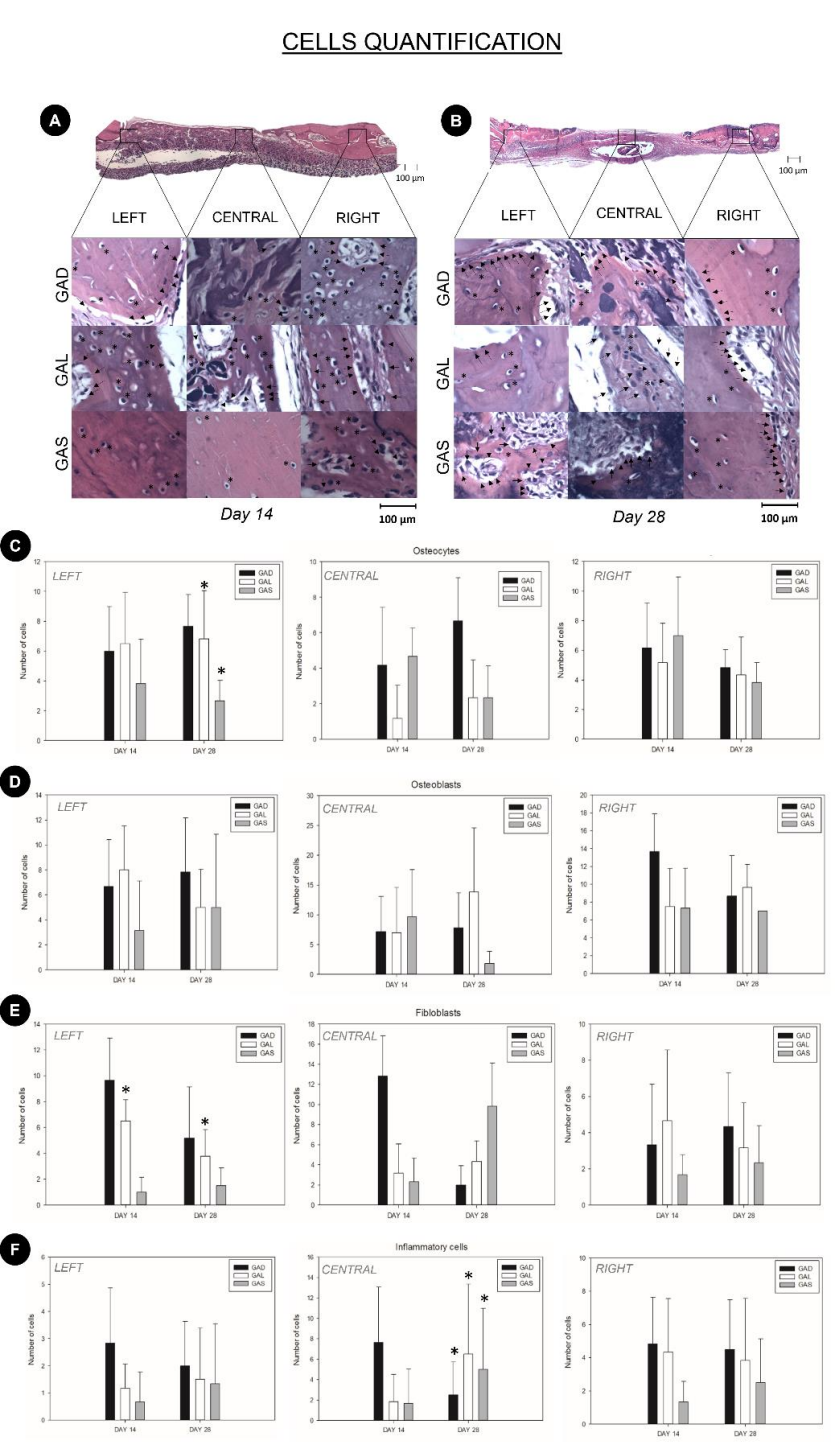
(A) Panoramic image with a 4x magnification representing the cell counting sites, left stump, central area, and right stump. 14-day experimental period. (H&E staining, original magnification 1000x). Representing osteoblasts in the images with arrows and osteocytes with *. (B) Panoramic image with a 4x magnification representing the cell counting sites, left stump, central area, and right stump. 14-day experimental period. (H&E staining, original magnification 1000x). Representing osteoblasts in the images with arrows and osteocytes with *. (C) Graphical representation of osteocyte count values for the experimental groups GAD, GAL, and GAS at 14 and 28 days postoperatively, in the left stump, central area, and right stump regions. * GAL and GAS at 28 days showed increasing values (p<0.05). (D) Graphical representation of osteoblast count values for the experimental groups GAD, GAL, and GAS at 14 and 28 days postoperatively, in the left stump, central area, and right stump regions. (E) Graphical representation of fibroblast count values for the experimental groups GAD, GAL, and GAS at 14 and 28 days postoperatively, in the left stump, central area, and right stump regions. * GAL at 14 and 28 days showed increasing values (p<0.05). (F) Graphical representation of inflammatory cell count values for the experimental groups GAD, GAL, and GAS at 14 and 28 days postoperatively, in the left stump, central area, and right stump regions. * GAL and GAS at 28 days showed increasing values (p<0.05).

Cells were counted in the right stump, central area and left stump (Figure 2 - A and B), as shown in the graphs (Figure 2 - C, D, E and F).

### Osteocytes

GAS group showed a decrease in the number of osteocytes compared to groups GAD (p<0.05) and GAL (p<0.05). Although no difference was observed at 14 days (p>0.05), at 28 days only the GAS group showed higher values than GAD (p=0.05).

For the osteocyte count in the right stump, all evaluation parameters revealed similar results (p>0.05; Two-way ANOVA) (Figure 2- C).

### Osteoblasts

The number of osteoblasts in the left stump, central area and right stump was similar, regardless of the parameter under analysis (p>0.05; Two-way ANOVA) (Figure 2- D).

### Fibroblasts

The number of fibroblasts in the left stump was higher only in group GAL from 14 to 28 days (p<0.05). The intergroup evaluation at 14 days showed no difference between GAL and GAD, whereas the other comparisons did reveal differences. However, after 28 days there were no significant group differences.

The number of fibroblasts in the central area and right stump was similar for all groups, regardless of the period analyzed (p>0.05, Two-way ANOVA) (Figure 2-E).

### Inflammatory cells

The number of inflammatory cells in the left and right stumps did not show a statistically significant difference in the ANOVA test. In the central area, there was a significant statistical difference in relation to group x time p<0.05. At 14 days, The GAS group did not differ from GAD, but the number of inflammatory cells was greater in GAL. At 28 days, both groups GAL and GAS presented a higher number of cells than GAD (Figure 2- F).

### Immunohistochemistry

*CD31*

**Figure 3:**
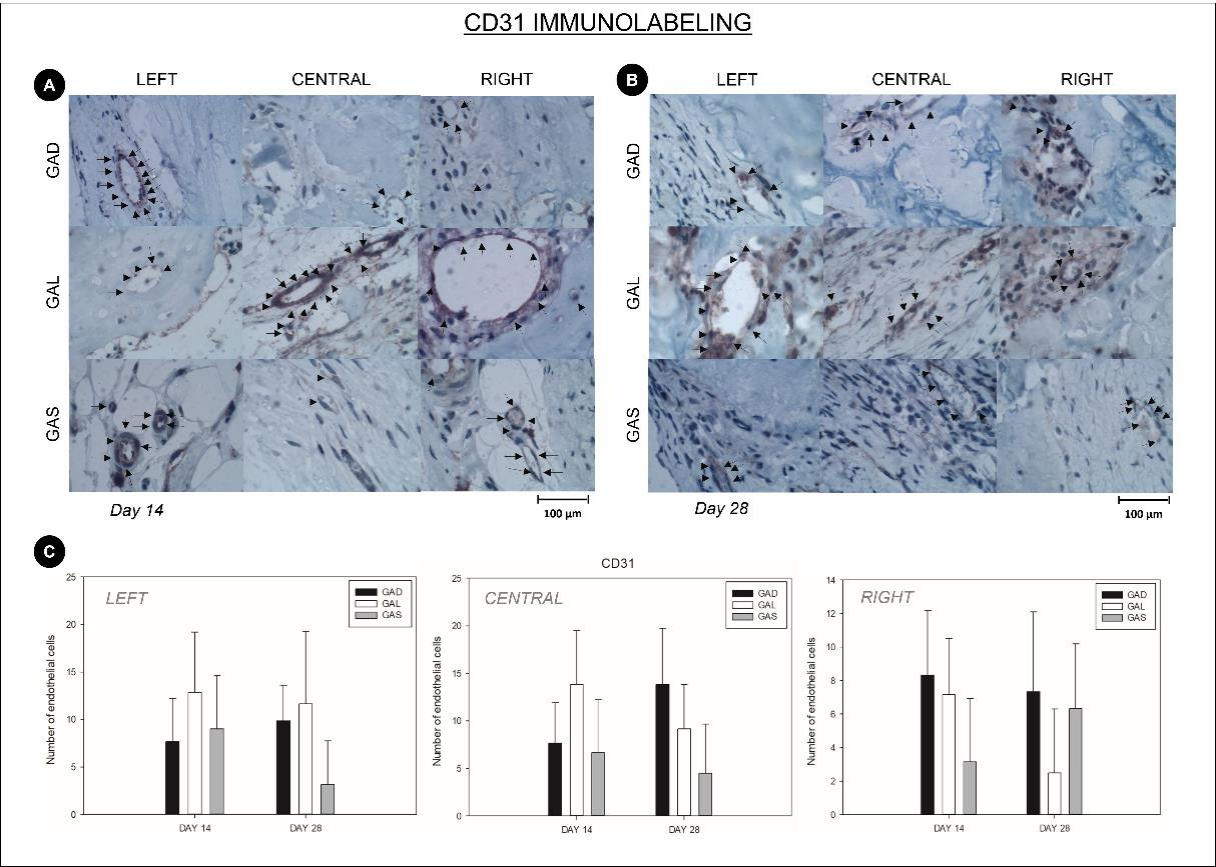
(A) Representative images of the immunohistochemical analysis of CD31 at 14 days. Black arrows indicating endothelial cells positive for the staining (1000X magnification, CD31 immunohistochemical analysis). (B) Representative images of the immunohistochemical analysis of CD31 at 28 days. Black arrows indicating endothelial cells positive for the staining (1000X magnification, CD31 immunohistochemical analysis). (C) Graphical representation of the number of endothelial cells with positive CD31 staining for the experimental groups GAD, GAL, and GAS at 14 and 28 days postoperatively. * The number of marked endothelial cells showed no statistically significant differences (p<0.05).

Immunohistochemical analysis did not revealed a statistically significant difference in endothelial cells positive for the CD31 marker in any of the evaluated locations or analyzed periods (14 and 28 days) (p>0.05; Two-way ANOVA) (Figure 3).

### Osteocalcin (osteoblasts)

**Figure 4:**
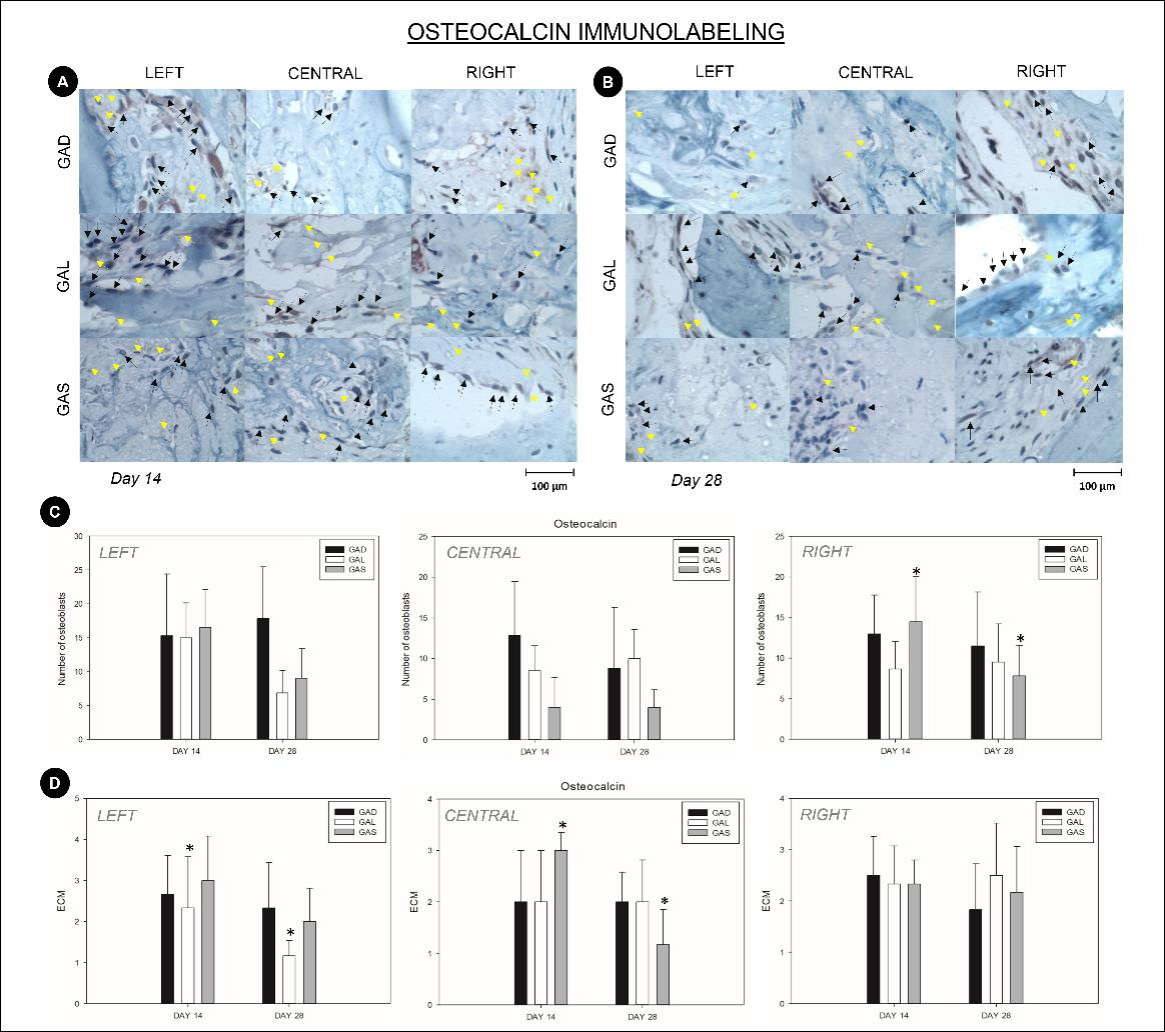
(A) Representative images of the immunohistochemical analysis of osteocalcin at 14 days. Black arrows indicating osteoblasts positive for the staining and yellow arrows indicating ECM positive for the staining (1000X magnification, osteocalcin immunohistochemical analysis). (B) Representative images of the immunohistochemical analysis of osteocalcin at 28 days. Black arrows indicating osteoblasts positive for the staining and yellow arrows indicating ECM positive for the staining (1000X magnification, osteocalcin immunohistochemical analysis). (C) Graphical representation of the number of osteoblasts with positive osteocalcin staining for the experimental groups GAD, GAL, and GAS at 14 and 28 days postoperatively. *GAS at both 14 and 28 days showed increased values with (p<0.05). (D) Graphical representation of the ECM score with positive osteocalcin staining for the experimental groups GAD, GAL, and GAS at 14 and 28 days postoperatively. *GAS at 28 days showed increased values (p<0.05).

The count of osteoblasts with positive OCN staining in the left and right stumps was similar across the analyzed groups and periods (p>0.05; Two-way ANOVA). In the central area, the count of osteoblasts positively labeled with OCN showed greater immunostaining at 14 days than at 28 days only in the GAS group (p<0.05; Holm-Sidak). The other group comparisons revealed similar results (p>0.05) (Figure 4-A,B and C).

### Osteocalcin (ECM)

ECM marking with the OCN marker on the left stump showed that only group GAL showed a decrease in immunostaining from day 14 to 28 (p<0.05; Holm-Sidak). The other group comparisons showed similar results (p>0.05).

In the central area, the ECM labeled with OCN showed lower immunostaining in the chronological evolution only for the GAS group (p<0.001; Holm-Sidak). The other group comparisons generated similar results (p>0.05) (Figure 4- A, B and D).

## Discussion

The investigation proposed by this study revealed that systemic administration of atorvastatin led to a significant increase in bone neoformation after critical defects in rat calvaria. The GAD and GAL groups did not present a significant increase in the area of neoformed bone, while Figures 1-B and 2-A showed an increase in the number of osteocytes in the extracellular matrix, as well as active osteoblasts at the margin of the region undergoing repair in the GAS group.

These findings indicate that systemically administered atorvastatin had a more significant osteogenic effect, contributing to bone neoformation. Furthermore, local applications with AD (distilled water) and AL (local application of atorvastatin) showed a similar neoformed bone area (NBA) at 14 and 28 days postoperatively.

Quantitative results also suggested that systemic administration linearly reduced the residual defect over time, leading to bone neoformation for more effective closure of the defect. This may be related to the administration route of the drug. Through the systemic route, it is easier to standardize the amount of drug absorbed by the body. Previous studies have shown that there is a difference between the forms of drug administration, in addition to difficulty in establishing the ideal dosage or maximum dosage for the drug's effect when applied locally.^14^ ^,^ ^18^ ^,^ ^19^ ^,^ ^25^

The literature mentions the use of statins in animal studies, in which standardization is not noted, with wide variations ranging from 0.1 to 120 mg/kg^1^ ^,^ ^13^ ^,^ ^18^ ^,^ ^24^ ^,^ ^25^. In addition to the dosage, the present study differs regarding the type of statin. Most studies have employed simvastatin, while a few experimental studies used atorvastatin. Atorvastatin is used in most clinical studies^26^ ^,^ ^27^ in doses ranging from 10 to 80 mg/day, which makes it difficult to compare findings in the literature with the results obtained in this study. It is important to highlight that atorvastatin was used because it has fewer side effects than simvastatin. Therefore, new generations of statins have been investigated in recent studies^10^ ^,^ ^11^.

In this context, the membrane and soft tissue area were not influenced by atorvastatin, and no local side effects like muscle necrosis, as reported by Shahrezaee et al.^25^ in their experimental study, were noted.

On the 14th day post-surgery, NBA in treatment with systemic atorvastatin was significantly higher in the GAS group than in the other groups (GAL and GAD) (p<0.05). Moreover, in a linear manner, the residual bone defect with systemic atorvastatin use was significantly smaller than in groups GAL and GAD after 14 days (p<0.05; p<0.05, respectively). Reaching different results, Xu, X.C. et al.^27^, have demonstrated that bone gains were significant when using local simvastatin 0.8 mg/0.05 mL to treat periodontal defects.^27^ This divergence may be justified by the fact that their study used a different substance at a different dosage, as well as a difference in the magnitude of the defects.

Regarding histomorphology, through cell quantification it is possible to affirm that there was no increase in osteoblasts, fibroblasts and osteocytes in some of the analyzed regions. With reference to the level of cell proliferation, atorvastatin, in both application forms, behaved similarly to distilled water, showing only an increase in the number of cells at some points, in line with the findings of Shahrezaee et al.^25^

The inflammatory cell count showed statistically significant differences in the central area, being the critical area of the defect. An increase was noted after 28 days for both routes of administration. Although inflammation is a part of the bone repair process, for the 5 mm defect in rat calvarias, 28 days is a long period of repair to present an abundant inflammatory process. These findings are inconsistent with the anti-inflammatory properties suggested by some authors.^25^ However, the results of the present study indicate that the increase in inflammation implies a presence of repair cells which, given the increased area of neoformed bone, are conducive to closing defects in the GAS group.

Considering the limitations of this study, future investigations should be realized to confirm the biological findings presented here, focusing on the marking of inflammatory cells like M1 and M2 macrophages and other interleukins.

Regarding CD31 immunostaining, there was no marked vascular proliferation in the presence of atorvastatins, unlike previous studies that had attributed a greater endothelial proliferation and an increased anti-inflammatory effect to statins.^28^ ^,^ ^29^

The CD31 used in this study showed positive staining only in endothelial cells and did not demonstrate an expression of proteins in differentiated and differentiating osteoblasts. A more specific study which employed a vascular and endothelial growth factor (VEGF) noted an increased expression of this marker in osteoblasts through reduced prenylation of proteins.^29^

A study using cell culture attributed an angiogenic property to the statin by inducing the production of nitric oxide and inhibiting the apoptosis of endothelial cells.^29^ Since theirs was an in vitro study, there are limitations when comparing these data with the results of the present study. However, it was noted that the presence of atorvastatin did not promote anti-inflammatory action and did not significantly induce angiogenesis. The increase in inflammatory cells did not interfere with bone neoformation, given that the presence of inflammatory cells is necessary for repair to occur and as previously discussed, some inflammatory cells can contribute to bone repair. Regarding newly formed blood vessels in histology, these proved to be sufficient to promote osteogenesis.

The decrease in OCN labeling in osteoblasts and in the ECM at 14 days in the GAS group complements the histometric findings. These revealed an increase in NBA, in corroboration with a clinical study where atorvastatin was administered systemically in cases of osteoporosis.^26^ The study by Yue, X. et al.^16^, showed the most effective results of local administration.Regarding the osteogenic benefit of statins, other studies have shown favorable results, as well as greater osteoblast differentiation.^1^ ^,^ ^18^ ^,^ ^13^

Still to prove the positive effects of atorvastin on bone repair, data on degradation of the collagen membrane and NBA have shown that the membrane, even in a critical defect, can conduct bone neoformation and increasing the repair process, as was also observed in a study previous.^12^ Furthermore, systemic atorvastin application in particular promoted greater osteogenesis.

This study has encountered some limitations, which were also mentioned in the literature^18^ ^,^ ^14^ ^,^ ^19^ ^,^ ^25^, such as establishing the maximum effective dose of systemic and local statins. Another limitation consists of the use of only one statin, in this case atorvastatin, which was chosen because it is regularly used in the treatment of hypercholerostemia and has fewer side effects. Although different statins share a structural similarity, they differ in solubility^20^. These characteristics are directly related to absorption and may vary according to the chosen route of administration^19^. This can explain the absence of significant data regarding the pattern of bone formation in the GAL group. In addition to solubility, statins have a low molecular weight that enables their quick release.

The promising results of this study encourage the development of future studies to determine the most effective dose of atorvastatin for osteogenesis; another proposal is to compare different statins to establish the most effective one. Furthermore, modifications to the carrier agent need to be evaluated to improve availability at the repair site, including the collagen membrane and the three-dimensional distribution of the drug.

## Conclusion

The results showed that systemic atorvastatin demonstrated enhanced osteogenesis in critical calvaria defects in rats, suggesting its efficacy in promoting bone regeneration with an anti-inflammatory effect.
